# Delayed standard care in diabetic foot ulcers: the roles of initial response pathways and non-professional health information

**DOI:** 10.3389/fpubh.2026.1905489

**Published:** 2026-09-25

**Authors:** Wei Shan, Qian Huang, Lin Qiao, Hui Chen, Hao Yan

**Affiliations:** Department of Interventional Vascular Surgery, General Hospital of Northern Theater Command, Shenyang, Liaoning, China

**Keywords:** amputation decision-making, care-seeking behavior, delayed standard care, diabetic foot ulcer, foot ulcer, health communication, health literacy, non-professional health information

## Abstract

**Background:**

Delayed entry into standard diabetic foot care may reflect non-standard initial actions, inaccurate risk appraisal, and exposure to unreliable health information. Practical concerns about life after amputation may further postpone treatment decisions, but these modifiable processes remain insufficiently characterized.

**Objective:**

To examine initial response pathways, non-professional health information exposure, and post-amputation living-support concerns in relation to delayed care, disease severity, limb outcomes, and amputation decision-making.

**Methods:**

A single-center prospective explanatory mixed-methods study included 400 adults with diabetic foot ulcers from August 2024 to February 2026. Questionnaires and records assessed response pathways, information exposure, delay >7 days, clinical outcomes, and amputation concerns. Logistic regression estimated adjusted associations. Interviews with 16 purposively selected participants explained quantitative patterns, and findings were integrated.

**Results:**

Delayed care occurred in 215 patients (53.8%), and 206 (51.5%) reported non-professional information exposure. Relative to direct standard care, non-specialist care (OR 2.03, 95% CI 1.07–3.85) and home-based or non-standard care (OR 4.61, 95% CI 2.35–9.04) were associated with delay; the information-exposure score was also associated (OR 1.59, 95% CI 1.30–1.94). Delay was associated with Wagner grade 3–5 disease (OR 2.18, 95% CI 1.43–3.32) and amputation (OR 1.85, 95% CI 1.11–3.08). Among 102 amputated patients with complete decision data, 54 (52.9%) delayed the decision; living-support concerns were associated with delay (OR 2.35, 95% CI 1.45–3.81). Interviews linked reduced urgency, competing information, self-treatment, and anticipated dependence or caregiving burden to these patterns.

**Conclusion:**

Delayed care reflected risk under-recognition, non-standard action, and competing information rather than inaction. Warning-sign education, service guidance, critical appraisal of health information, and structured amputation decision support may improve timely care.

## 1 Introduction

Diabetic foot ulceration is a major complication of diabetes and is associated with disability, mortality, and substantial healthcare burden (1–4). Timely specialist assessment, debridement, infection management, vascular evaluation, and offloading are important for limiting progression and amputation (2, 4–9). Yet the interval before standard care often contains active but inadequate responses, including observation, self-dressing, topical remedies, or repeated attendance at non-specialist facilities. Delayed care should therefore be examined as a care pathway, not simply as failure to seek help.

Previous studies mainly focused on demographic characteristics, diabetes knowledge, and socioeconomic determinants of delayed care (10–22). However, little is known regarding how patients’ initial response pathways, exposure to non-professional health information, and concerns regarding post-amputation living support interact to influence delayed standard care. This gap is important because these variables represent potentially modifiable stages in risk appraisal, information evaluation, entry into appropriate care, and acceptance of a major treatment decision (23–34).

This study therefore examined whether initial response pathways and non-professional health information exposure were associated with delayed standard care and adverse limb outcomes, and whether living-support concerns were associated with delayed amputation decisions. We hypothesized the relationships shown in Figure 1 as an observational framework, not a causal model. A prospective explanatory mixed-methods design was used so that interviews could explain why the quantitative associations occurred and identify modifiable intervention targets.

**Figure 1:**
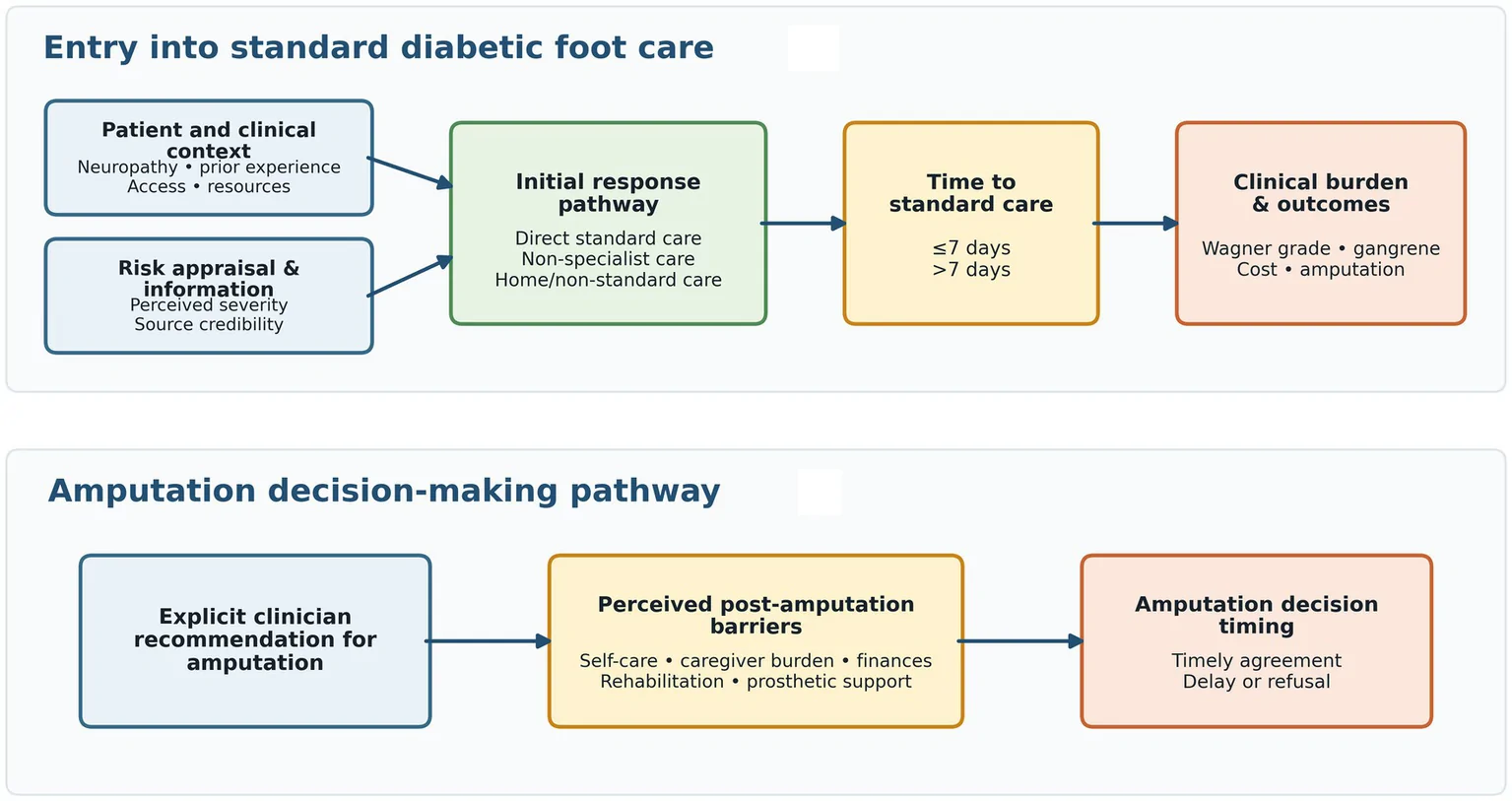
Conceptual framework of delayed standard care and amputation decision-making. Arrows indicate hypothesized, non-causal associations.

## 2 Methods

### 2.1 Study design

This study used a single-center prospective observational mixed-methods design, with quantitative research as the main component and embedded explanatory qualitative interviews. Reporting was guided by relevant mixed-methods, observational, and qualitative reporting recommendations (35–37). Quantitative findings informed interview sampling and the interview guide, and the two strands were integrated through a joint display (38). The study was conducted from August 2024 to February 2026. The study was approved by the Ethics Committee of the General Hospital of the Northern Theater Command of the Chinese People’s Liberation Army (approval No. 2024(112)). All participants provided written informed consent before participation.

### 2.2 Study population

Patients with diabetic foot ulcers treated in diabetic foot-related departments of the General Hospital of the Northern Theater Command of the Chinese People’s Liberation Army were recruited. The relevant departments included endocrinology, wound repair, vascular surgery, orthopedics, and other clinical departments responsible for diabetic foot care.

The inclusion criteria were as follows: (1) age ≥18 years; (2) diagnosis of type 2 diabetes mellitus; (3) diagnosis of diabetic foot ulcer, with or without infection, gangrene, or ischemia, or need for specialist wound management; (4) Wagner grade 1–5; (5) clear consciousness and ability to complete the questionnaire survey or interview; and (6) voluntary participation with signed informed consent.

The exclusion criteria were as follows: (1) foot ulcer, wound, or limb gangrene not caused by diabetes; (2) severe cognitive impairment, mental disorder, disturbance of consciousness, or language communication impairment; (3) critical illness preventing completion of the survey; (4) inability to recall the onset time of foot symptoms, initial response process, or care-seeking process; and (5) refusal to participate or withdrawal during the study.

### 2.3 Sample size

The quantitative sample size was estimated based on the expected prevalence of delayed standard care. The sample size formula for a proportion was *n* = *Z*^2^*α*/2 × *p*(1 − *p*)/*d*^2^. With a two-sided α of 0.05, *Zα*/2 was 1.96. Because the prevalence of delayed standard care in the target population was not fully known, p was set at 0.50 for a conservative estimate, and the allowable error d was set at 0.05. The minimum effective sample size was calculated to be 385. Considering the stability requirements of multivariable logistic regression analysis, the planned effective quantitative sample size was 400.

Purposive sampling was used for the qualitative interviews, with 15–20 participants selected from the quantitative sample. Interviewees included patients with marked delayed standard care, those who had self-managed or received non-specialist care, those exposed to non-professional health information, and patients who accepted an amputation recommendation promptly or initially hesitated or declined but subsequently underwent amputation. The qualitative sample size was determined based on data saturation.

### 2.4 Study instruments and data collection

#### 2.4.1 General information questionnaire

A researcher-designed questionnaire was used to collect age, sex, marital status, educational level, residence, employment status, monthly household income, type of medical insurance, living alone, presence of a primary caregiver, family caregiving ability, smoking history, and drinking history.

#### 2.4.2 Clinical data extraction form

Clinical data were extracted from the medical record system, including duration of diabetes, previous diabetic foot history, previous amputation history, hypertension, coronary heart disease, cerebrovascular disease, chronic kidney disease, peripheral neuropathy, and peripheral arterial disease. Laboratory indicators included glycated hemoglobin (HbA1c), white blood cell count, C-reactive protein, hemoglobin, albumin, and creatinine. Diabetic foot-related data included ulcer site, ulcer area, infection, gangrene, osteomyelitis, ischemia, Wagner grade, SINBAD score, number of debridements, vascular intervention, amputation, amputation level, length of hospital stay, hospitalization cost, and wound outcome at discharge.

#### 2.4.3 Initial response pathway questionnaire

A structured questionnaire was used to collect the treatment process from the first recognition of foot abnormalities to the receipt of standard care. Foot abnormalities included foot ulceration, blisters, black discoloration, purulent discharge, pain, burns, shoe-related injuries, or other abnormal manifestations. For statistical analysis, initial response pathways were combined into three categories according to the actual sample distribution: direct standard care, non-specialist medical care, and home-based or non-standard care.

#### 2.4.4 Exposure to non-professional health information item set

A researcher-designed item set was used to assess whether patients were exposed to diabetic foot-related health information from social media platforms, short-video platforms, online patient communities, information forwarded by relatives or friends, commercial limb-salvage promotion, non-professional treatment advertisements, or other non-professional sources after noticing foot abnormalities. Each item was scored on a 5-point Likert scale from “strongly disagree” to “strongly agree.” The mean item score (range, 1–5) was used, with higher scores indicating greater reported influence from non-professional health information. The item set was revised after expert consultation and a pilot survey before formal use. Cronbach’s alpha for the item set was 0.922, and the scale-level content validity index based on the average method (S-CVI/Ave) was 0.922.

#### 2.4.5 Concerns about post-amputation living support item set

The post-amputation living-support item set was administered only to patients for whom the treating team documented an explicit recommendation for major or minor amputation. It was not administered to the remaining participants because the items were not clinically applicable and would have elicited hypothetical responses. The content included reduced self-care ability, long-term dependence on family care, increased financial burden, increased caregiving burden, insufficient rehabilitation and prosthetic support, reduced social participation, and decreased quality of life. Each item was scored on a 5-point Likert scale. The mean item score (range, 1–5) was used as an exploratory index; higher scores indicated greater concern. The item set was developed based on literature review and clinical interview experience and was revised after expert consultation and a pilot survey before formal use. Cronbach’s alpha was 0.855, and S-CVI/Ave was 0.915.

### 2.5 Definitions of main variables

#### 2.5.1 Delayed standard care

Delayed standard care time was defined as the interval between the patient’s first recognition of the current diabetic foot-related abnormality and the first receipt of standard diabetic foot care. Foot abnormalities included foot ulceration, ulcer, ruptured blister, black discoloration, purulent discharge, progressive redness, swelling and pain, gangrene, or other manifestations clearly indicating diabetic foot risk. Standard diabetic foot care included specialist diabetic foot assessment, standard debridement, anti-infective therapy, vascular assessment, offloading, comprehensive inpatient treatment, or a systematic treatment plan developed by a medical team with diabetic foot care capability (2, 5–9). In the primary analysis, delayed standard care was defined as a delay of >7 days between first recognizing foot abnormalities and first receiving standard diabetic foot care. A delay of >30 days was defined as severe delayed standard care. Delay time was further categorized as ≤24 h, 2–7 days, 8–30 days, and >30 days. In sensitivity analyses, ≥24 h and >30 days were used as alternative thresholds to examine the stability of the results.

#### 2.5.2 Rationale for the delay thresholds

The term standard care referred to the type and provider of care, not to a 24-h treatment duration. The time categories (≤24 h, 2–7 days, 8–30 days, and >30 days) were prespecified to describe progressively longer intervals from first recognition of a foot abnormality to first receipt of standard care; they were not intended as a universal clinical definition. The ≤24-h category represented the earliest-care stratum. NICE recommends immediate referral for limb- or life-threatening diabetic foot problems and referral within one working day for other active problems (39), while IWGDF/IDSA recommends urgent surgical consultation and, when indicated, early surgery within 24–48 h for moderate or severe infection (6). These guidelines support clinical urgency but do not define a universal patient-level delay threshold. The >7-day primary outcome separated care obtained within the first week from care obtained after 1 week. A one-week boundary has been used to categorize ulcer duration in the multicenter Eurodiale cohort (40) and care-seeking delay in Chinese research (41), and a recent cohort used >7 days to define delayed presentation (42). Published definitions nevertheless remain heterogeneous (10, 43). The >30-day category denoted severe delay; a four-country European study found that referral occurred after more than 1 month or at an unknown interval in approximately 48% of cases (44). Sensitivity analyses using ≥24 h and >30 days assessed robustness to earlier and more stringent definitions.

#### 2.5.3 Delayed amputation decision-making

Delayed amputation decision-making was assessed only after the treating team had documented an explicit recommendation for major or minor amputation. It referred to delay in the patient’s acceptance of or consent to the recommended procedure, rather than clinical or scheduling delay after consent. Decision time was measured from the first explicit clinician recommendation for amputation to the signing of surgical consent or receipt of amputation surgery. Delay was defined as an interval of >7 days, documented initial refusal, repeated requests for continued limb salvage, or postponement of consent that delayed eventual surgery. Initial refusal denoted refusal followed by subsequent acceptance and amputation during the observation period; no patient with a documented amputation recommendation remained non-amputated.

#### 2.5.4 Clinical outcomes

The primary outcomes included: (1) severe diabetic foot at admission, defined as Wagner grade 3–5; (2) amputation; and (3) major amputation. Major amputation was defined as amputation above the ankle joint, while minor amputation was defined as amputation at or below the ankle joint or toe amputation. Secondary outcomes included infection, gangrene, osteomyelitis, ischemia, number of debridements, vascular intervention, length of hospital stay, hospitalization cost, transfer to the intensive care unit, in-hospital death, wound healing or improvement at discharge, and readmission, reoperation, recurrence, or death within 1 month after discharge.

### 2.6 Data collection procedures

After admission or consultation, patients were screened by uniformly trained researchers. Participant disposition was documented sequentially at four stages: eligibility screening, consent, completion of the prespecified core dataset, and inclusion in the quantitative analysis. Reasons for non-eligibility, refusal after eligibility confirmation, and exclusion after consent because of incomplete core data were recorded as mutually exclusive categories. When a patient’s condition was stable and the patient was able to cooperate with the survey, the researchers explained the study purpose, data collection content, privacy protection measures, and right to withdraw, and obtained written informed consent. All 400 participants included in the final quantitative cohort completed the core questionnaire modules on general information, symptom onset, initial response pathway, and non-professional health information exposure.

Researchers used structured questionnaires to collect general information, onset time of foot symptoms, initial response pathways, and exposure to non-professional health information. The amputation-specific module was administered conditionally, only after a documented recommendation for major or minor amputation. For these patients, the time of the first amputation recommendation, whether the patient agreed immediately, whether the patient initially refused or hesitated, the duration of any delay, and the main concerns were recorded. All 108 patients with a documented recommendation ultimately underwent amputation during the observation period; complete recommendation-timing, decision-process, and concern-score data were available for 102 patients, while six had incomplete decision-making data.

Clinical data were extracted from the electronic medical record system, including diabetic foot severity, laboratory tests, treatment measures, and inpatient outcomes. At discharge, length of hospital stay, hospitalization cost, wound outcome, amputation status, and in-hospital death were recorded. After discharge, wound healing, readmission, reoperation, recurrence, and death were recorded through outpatient follow-up, telephone follow-up, or the medical record system.

### 2.7 Qualitative interviews

The qualitative component used semi-structured interviews. After preliminary quantitative analysis, 16 patients were purposively sampled from the quantitative study participants to explain the principal patterns and capture contrasting experiences. Selection considered marked delayed versus timely care, direct standard versus non-specialist or home-based care, reported versus no non-professional health information exposure, and, among patients with an explicit amputation recommendation, prompt acceptance versus initial hesitation or refusal followed by eventual amputation. No new themes or important information emerged by the 16th interview, indicating data saturation.

The interviews focused on patients’ early judgment after detecting foot abnormalities, initial response methods, the influence of non-professional health information on care-seeking behavior, reasons for not receiving standard care promptly, reactions when clinicians recommended amputation, concerns about self-care, family caregiving, economic burden, rehabilitation support, and quality of life after amputation, and needs for early health education and amputation decision support.

### 2.8 Quality control

Before the study, all investigators received standardized training on inclusion and exclusion criteria, variable definitions, questionnaire completion, medical record extraction, and interview procedures. A pilot survey was conducted before formal data collection, and the structured questionnaire was revised according to patient comprehension and feedback. The researcher-designed item sets were evaluated for content validity through expert consultation. During data collection, researchers cross-checked key time points using patient reports, information from family members, and medical records to reduce recall bias. All quantitative data were coded uniformly and double-checked. Qualitative interviews were conducted by trained researchers, and audio recordings were transcribed promptly and de-identified. Interview transcripts were independently coded by two researchers; disagreements were resolved through discussion and, when necessary, adjudication by a third researcher.

### 2.9 Statistical analysis

SPSS 26.0 was used for statistical analysis. Continuous variables were expressed as means ± standard deviations or medians with interquartile ranges according to their distributions. Between-group comparisons were performed using the t test, analysis of variance, Mann–Whitney *U* test, or Kruskal–Wallis *H* test as appropriate. Categorical variables were expressed as frequencies and percentages, and between-group comparisons were performed using the *χ*^2^ test or Fisher’s exact test.

Descriptive analyses were first conducted for demographic characteristics, clinical features, initial response pathways, exposure to non-professional health information, delayed standard care, concerns about post-amputation living support, delayed amputation decision-making, and clinical outcomes. Group comparisons were then performed according to delayed standard care status, initial response pathway, and delayed amputation decision-making status.

The dimensionality and preliminary structural validity of the two researcher-designed item sets were examined separately using exploratory factor analysis (EFA). Sampling adequacy was assessed using the Kaiser–Meyer–Olkin (KMO) statistic and Bartlett’s test of sphericity. Factors were extracted by principal axis factoring from Pearson item-correlation matrices. The number of factors was determined using the scree plot and parallel analysis based on 1,000 random correlation matrices with the 95th-percentile criterion. An oblique rotation was prespecified if more than one factor was retained; no rotation was applied when a one-factor solution was selected. Factor loadings, communalities, corrected item–total correlations, item-level measures of sampling adequacy, and Cronbach’s alpha were examined.

Multivariable logistic regression was used to analyze factors associated with delayed standard care, severe diabetic foot at admission, and amputation. Variables with clinical significance or *p* < 0.10 in univariate analysis were included in multivariable models. In the subgroup of patients with a documented amputation recommendation and complete recommendation-timing, decision-process, and living-support concern data, multivariable logistic regression was used to examine the association between concerns about post-amputation living support and delayed amputation decision-making. Continuous outcomes such as length of hospital stay and hospitalization cost were analyzed using linear regression, generalized linear models, or non-parametric methods according to data distribution. Sensitivity analyses were performed using alternative thresholds for delayed standard care. All statistical tests were two-sided, and *p* < 0.05 was considered statistically significant. Multivariable logistic regression results were reported as odds ratios (ORs) and 95% confidence intervals (CIs). The regression analyses were used to estimate adjusted associations rather than to develop a clinical prediction model.

Multicollinearity was assessed using tolerance and variance inflation factors (VIFs). Model fit was examined using the Hosmer–Lemeshow goodness-of-fit test, and model explanatory performance was summarized using Nagelkerke’s pseudo-*R*-squared. Discrimination was evaluated using the area under the receiver operating characteristic curve (AUC), with 95% confidence intervals obtained from 2,000 bootstrap resamples. These diagnostics were applied to the same four logistic regression models used for the main association analyses. Because the models were explanatory rather than predictive, the diagnostic measures were used as supportive checks and not to claim clinical prediction performance.

Missing data were handled using complete-case analysis for the relevant analysis; no multiple imputation was performed, and missingness was not assumed to be completely at random. Six of the 108 patients with a documented amputation recommendation had incomplete amputation decision-making data and were excluded only from the corresponding subgroup analysis, leaving 102 complete cases. Potential selection bias arising from complete-case analysis was considered when interpreting the findings.

### 2.10 Qualitative data analysis

Qualitative data were analyzed using thematic analysis. Researchers repeatedly read the interview transcripts to become familiar with the data; open coding was performed for statements related to delayed standard care, non-professional information influence, initial response pathways, amputation decision conflict, and concerns about post-amputation living support. Similar codes were then grouped into subthemes, and core themes were extracted through further integration. Integration occurred through connecting (quantitative findings informed participant selection), building (the findings informed the interview guide), and merging (quantitative estimates and qualitative explanations were compared in a joint display). Convergence, complementarity, and discordance were considered before integrated interpretations were developed (Figure 2 and Table 1) (38). The integration focused on how non-professional health information influenced initial response pathways, why patients delayed entry into standard care, and how concerns about post-amputation living support influenced amputation decisions.

**Figure 2:**
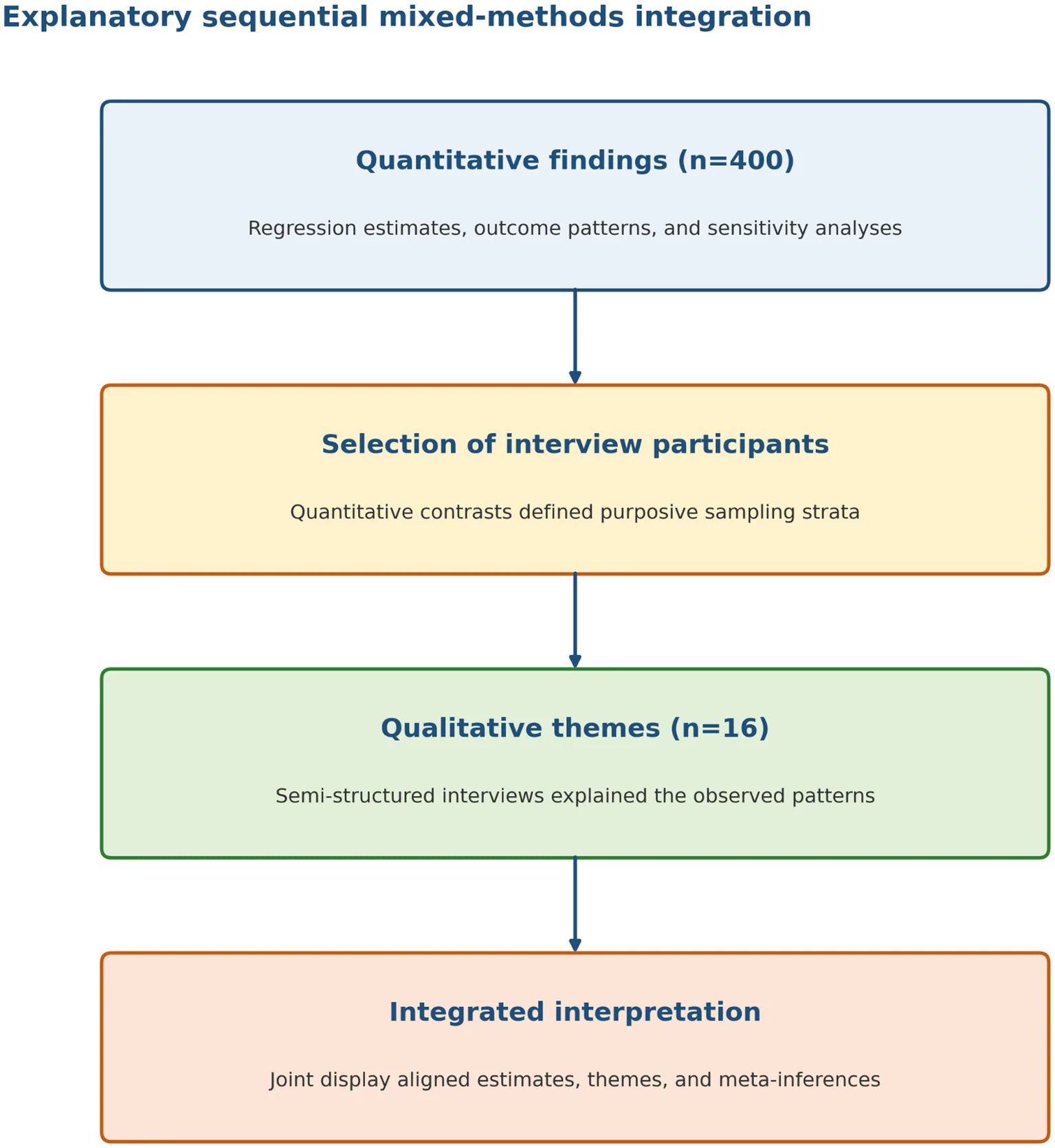
Explanatory mixed-methods integration process. Quantitative findings informed purposive sampling and the interview guide; the two strands were then merged through a joint display to generate integrated interpretations.

**Table 1:** Joint display of quantitative findings, qualitative explanations, and integrated inferences.

Domain	Quantitative finding	Qualitative explanation	Integrated inference
Initial response pathway	Only 94/400 (23.5%) entered standard care directly; home-based/non-standard care: adjusted OR 4.61 (95% CI 2.35–9.04) for delay.	Painless or small wounds were treated as minor; self-dressing, pharmacy remedies, or nearby clinics felt proportionate and convenient.	Delay commonly reflected failure to escalate from an initial action, not complete absence of care-seeking. Education must specify both warning signs and where to go.
Non-professional health information	206/400 (51.5%) reported exposure; each 1-point score increase: adjusted OR 1.59 (95% CI 1.30–1.94) for delay.	Short videos, acquaintances, patient communities, and limb-salvage promotion provided reassuring anecdotes or remedies that supported continued self-treatment.	Competing information can alter perceived urgency and legitimize non-standard care. Critical appraisal and source-specific correction are actionable targets.
Delay and limb outcomes	Delayed care: adjusted OR 2.18 (95% CI 1.43–3.32) for Wagner grade 3–5 and OR 1.85 (95% CI 1.11–3.08) for amputation.	Patients often escalated only after swelling, black discoloration, drainage, odor, or functional decline made deterioration unmistakable.	Missed early assessment, offloading, infection control, or vascular evaluation offers a plausible pathway to accumulated tissue damage, although causality is not established.
Amputation decision-making	54/102 (52.9%) delayed acceptance before ultimately undergoing amputation.	Concerns centered on future dependence, burdening family, rehabilitation and prosthetic access, cost, and social participation – not only fear of surgery.	Risk communication alone may be insufficient. Decision support should include a concrete post-discharge, caregiving, rehabilitation, and financial plan.
Need for credible support	Sensitivity analyses retained the direction of the main associations across ≥ 24-h and >30-day thresholds.	After deterioration, participants expressed regret and requested earlier, credible, action-oriented guidance.	Interventions should combine credible digital information, clear routing to specialist care, caregiver involvement, and timely decision support.

### 2.11 Ethical considerations

This was an observational study that did not interfere with patients’ treatment plans or increase additional examination or treatment risks. Before study participation, researchers explained the study purpose, content, potential risks, benefits, confidentiality measures, and right to withdraw. Patients were included only after providing written informed consent.

All study data were managed using coded identifiers, and identifiable information such as names, identity card numbers, and telephone numbers was removed. Electronic data were password-protected and accessible only to research team members. Separate consent was obtained for audio recording of qualitative interviews, and recordings were de-identified after transcription. No personally identifiable information was presented in published results.

## 3 Results

### 3.1 Patient characteristics and clinical features

During the study period, 487 patients were screened. Sixteen were excluded before enrollment because they did not meet the eligibility criteria, including nine with severe cognitive impairment and seven for other prespecified reasons, leaving 471 eligible patients who were approached. Of these, 446 provided written informed consent and 25 declined, corresponding to a consent rate of 94.7%. Among the 446 consented participants, 46 did not provide complete prespecified core questionnaire or clinical data and were excluded from the complete-case cohort. The final quantitative analysis therefore included 400 participants, corresponding to an analytic completion rate of 89.7% among consented participants and an overall usable response rate of 84.9% among eligible patients approached (Figure 3 and Table 2).

**Figure 3:**
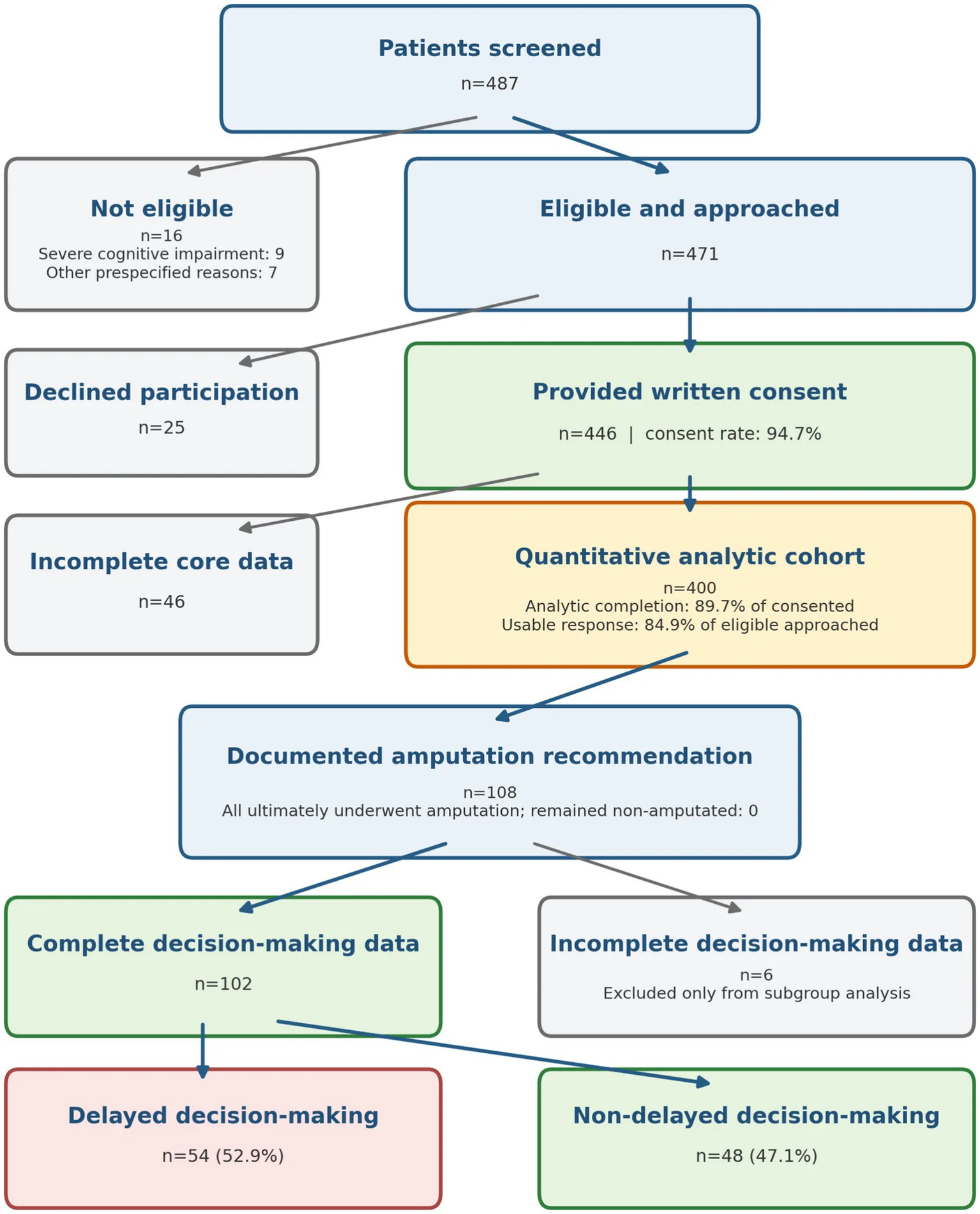
Participant flow and response rates. Participation rate was calculated among eligible patients approached; the complete-data response rate was calculated among participants who consented.

**Table 2:** Patient characteristics and clinical features.

Variable	Overall (*n* = 400)
Age, years	65 (56–73)
Male, *n* (%)	256 (64.0)
Educational level, *n* (%)	Primary school or below 68 (17.0); junior high school 132 (33.0); senior high school/technical secondary school 120 (30.0); college or above 80 (20.0)
Residence, *n* (%)	Urban 178 (44.5); rural 222 (55.5)
Monthly household income, *n* (%)	<3,000 yuan 58 (14.5); 3,000–7,000 yuan 226 (56.5); >7,000 yuan 116 (29.0)
Living alone, *n* (%)	88 (22.0)
Primary caregiver, *n* (%)	298 (74.5)
Duration of diabetes, years	11 (6–17)
Previous diabetic foot history, *n* (%)	114 (28.5)
HbA1c, %	8.4 (7.0–10.1)
Wagner grade, *n* (%)	Grade 1 48 (12.0); grade 2,172 (43.0); grade 3 96 (24.0); grade 4 66 (16.5); grade 5 18 (4.5)
Wagner grade 3–5, *n* (%)	180 (45.0)
Infection, *n* (%)	274 (68.5)
Gangrene, *n* (%)	98 (24.5)
Osteomyelitis, *n* (%)	85 (21.3)
Ischemia, *n* (%)	210 (52.5)
Amputation, *n* (%)	108 (27.0)
Major amputation, *n* (%)	38 (9.5)
Length of hospital stay, days	20 (12–36)
Hospitalization cost, yuan	27,800 (14,800–48,900)
Wound healing or improvement at discharge, *n* (%)	272 (68.0)

Exploratory factor analysis provided preliminary support for a one-factor structure for each researcher-designed item set. For the non-professional health-information item set (n = 400), sampling adequacy was high (KMO = 0.937; Bartlett’s chi-square = 1733.24, df = 21, *p* < 0.001). Parallel analysis supported retention of one factor, and the principal-axis solution accounted for 62.5% of the variance; factor loadings ranged from 0.763 to 0.829, and Cronbach’s alpha was 0.922. For the post-amputation living-support item set (*n* = 102), the KMO was 0.885 and Bartlett’s test was significant (chi-square = 251.86, df = 21, *p* < 0.001). Parallel analysis also supported retention of one factor, accounting for 46.3% of the variance; factor loadings ranged from 0.572 to 0.768, and Cronbach’s alpha was 0.855. These findings provide preliminary evidence of structural coherence but do not constitute independent validation of either item set.

### 3.2 Initial response pathways, exposure to non-professional health information, and delayed standard care

After first noticing foot abnormalities, 94 patients (23.5%) directly received standard care, 152 (38.0%) first received non-specialist medical care, and 154 (38.5%) adopted home-based or non-standard care. A total of 206 patients (51.5%) reported exposure to non-professional health information, including social media, short-video platforms, online patient communities, information forwarded by relatives or friends, and commercial limb-salvage promotion.

The median time from first noticing foot abnormalities to first receiving standard care was 8 (4–20) days. Using >7 days as the definition of delayed standard care, 215 patients (53.8%) experienced delayed standard care. Using >30 days as the definition of severe delayed standard care, 90 patients (22.5%) experienced severe delay. Compared with the non-delayed group, the delayed group had higher proportions of home-based or non-standard care and exposure to non-professional health information, as well as higher non-professional health information exposure scores. The delayed group also had higher proportions of Wagner grade 3–5 disease and gangrene at admission, a higher in-hospital amputation rate, longer hospital stays, and higher hospitalization costs (all *p* < 0.05), while the difference in infection was not statistically significant (Table 3).

**Table 3:** Comparison of patient characteristics and clinical outcomes between the delayed and non-delayed groups.

Variable	Non-delayed group (*n* = 185)	Delayed group (*n* = 215)	*p*-value
Age, years	64 (55–72)	67 (57–74)	0.08
Male, *n* (%)	122 (65.9)	134 (62.3)	0.46
Primary school or below, *n* (%)	26 (14.1)	42 (19.5)	0.15
Rural residence, *n* (%)	96 (51.9)	126 (58.6)	0.18
Monthly household income <3,000 yuan, *n* (%)	22 (11.9)	36 (16.7)	0.22
Living alone, *n* (%)	38 (20.5)	50 (23.3)	0.51
Duration of diabetes, years	10 (6–16)	12 (7–18)	0.09
Previous diabetic foot history, *n* (%)	58 (31.4)	56 (26.0)	0.24
HbA1c, %	8.2 (6.9–9.8)	8.6 (7.2–10.3)	0.07
Initial response pathway, *n* (%)			<0.001
Direct standard care	68 (36.8)	26 (12.1)	
Non-specialist medical care	66 (35.7)	86 (40.0)	
Home-based or non-standard care	51 (27.6)	103 (47.9)	
Exposure to non-professional health information, *n* (%)	74 (40.0)	132 (61.4)	<0.001
Non-professional health information exposure score	2.3 (1.5–3.1)	3.5 (2.7–4.3)	<0.001
Wagner grade 3–5, *n* (%)	64 (34.6)	116 (54.0)	<0.001
Infection, *n* (%)	124 (67.0)	150 (69.8)	0.56
Gangrene, *n* (%)	34 (18.4)	64 (29.8)	0.009
Amputation, *n* (%)	38 (20.5)	70 (32.6)	0.007
Major amputation, *n* (%)	12 (6.5)	26 (12.1)	0.06
Length of hospital stay, days	15 (8–27)	25 (14–42)	<0.001
Hospitalization cost, yuan	19,500 (11,200–35,800)	34,200 (18,200–57,800)	<0.001

### 3.3 Factors associated with delayed standard care and major clinical outcomes

Multivariable logistic regression analysis showed that, compared with direct standard care, non-specialist medical care and home-based or non-standard care were both associated with delayed standard care. Higher non-professional health information exposure scores were also independently associated with delayed standard care. Primary school education or below showed a positive trend toward association with delayed standard care, but did not reach statistical significance. Previous diabetic foot history was not significantly associated with delayed standard care. Further analysis showed that, after adjusting for age, sex, duration of diabetes, HbA1c, and previous diabetic foot history, delayed standard care was associated with Wagner grade 3–5 disease at admission and amputation. Sensitivity analyses using ≥24 h and >30 days as alternative thresholds showed that the main directions of association were consistent with the primary analysis. The associations were generally attenuated under the ≥24-h threshold and stronger under the >30-day threshold, particularly for home-based or non-standard care, non-professional health information exposure, Wagner grade 3–5 disease, and amputation (Supplementary Table 1; Figure 4; Table 4).

**Figure 4:**
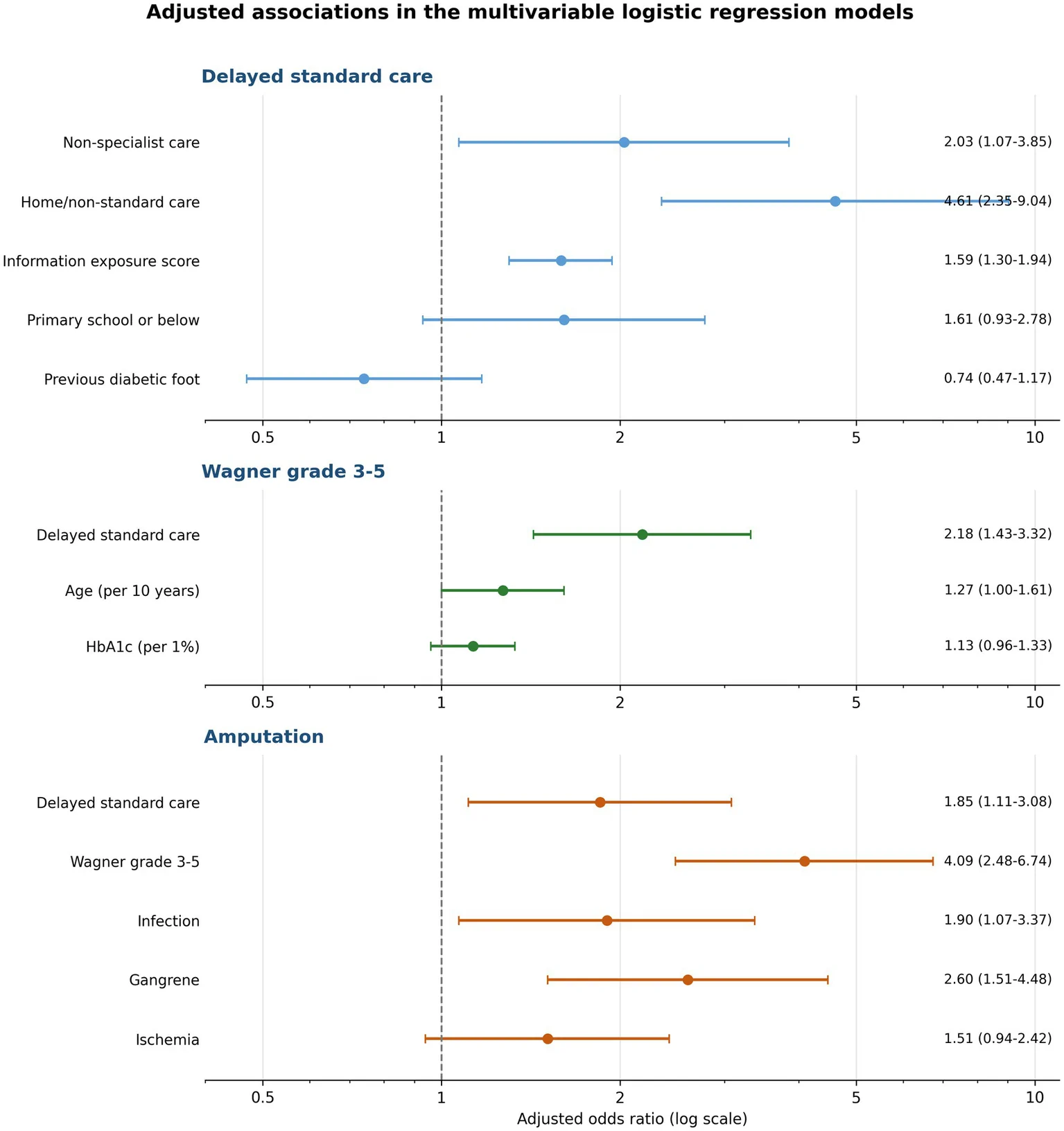
Forest plot of adjusted odds ratios (ORs) and 95% confidence intervals (CIs) from the three multivariable logistic regression models. The vertical dashed line denotes OR = 1.

**Table 4:** Multivariable logistic regression analysis of delayed standard care and major clinical outcomes.

Model	Outcome	Factor	OR	95% CI	*P*-value
Model 1	Delayed standard care	Non-specialist medical care (vs. direct standard care)	2.03	1.07–3.85	0.03
		Home-based or non-standard care (vs. direct standard care)	4.61	2.35–9.04	<0.001
		Non-professional health information exposure score (per 1 point)	1.59	1.30–1.94	<0.001
		Primary school or below (vs. junior high school or above)	1.61	0.93–2.78	0.09
		Previous diabetic foot history	0.74	0.47–1.17	0.20
Model 2	Wagner grade 3–5	Delayed standard care	2.18	1.43–3.32	<0.001
		Age (per 10 years)	1.27	1.00–1.61	0.05
		HbA1c (per 1%)	1.13	0.96–1.33	0.14
Model 3	Amputation	Delayed standard care	1.85	1.11–3.08	0.02
		Wagner grade 3–5	4.09	2.48–6.74	<0.001
		Infection	1.90	1.07–3.37	0.03
		Gangrene	2.60	1.51–4.48	0.001
		Ischemia	1.51	0.94–2.42	0.09

Only key variables are shown in the table. Model 1 included variables with clinical significance or p < 0.10 in univariate analysis. Model 2 was adjusted for age, sex, duration of diabetes, HbA1c, and previous diabetic foot history. Model 3 included delayed standard care, Wagner grade 3–5 disease, infection, gangrene, and ischemia. OR, odds ratio; CI, confidence interval.

### 3.4 Analysis of delayed amputation decision-making

During hospitalization, 108 patients received a documented recommendation for major or minor amputation, and all 108 ultimately underwent the recommended procedure, including 38 major and 70 minor amputations. No patient with a documented amputation recommendation remained non-amputated during the observation period. The amputation rate was higher in the delayed standard care group than in the non-delayed group [32.6% (70/215) vs. 20.5% (38/185), *p* = 0.007]. The proportion of major amputation was also higher in the delayed group [12.1% (26/215) vs. 6.5% (12/185)], although the difference did not reach statistical significance (*p* = 0.06).

Of the 108 patients with a documented amputation recommendation, 102 had complete data on the timing of the first recommendation, the subsequent decision-making process, and concerns about post-amputation living support. Six patients were excluded only from the corresponding subgroup analysis because of incomplete decision-making data. Among the 102 complete cases, 54 (52.9%) experienced delayed amputation decision-making before ultimately undergoing amputation. Patients with delayed decision-making had higher living-support concern scores than those without delayed decision-making [4.0 (3.3–4.6) vs. 2.6 (2.0–3.2), *p* = 0.001]. After adjustment for age, family caregiving support, and disease severity, a higher living-support concern score remained independently associated with delayed amputation decision-making (OR 2.35, 95% CI 1.45–3.81, *p* = 0.001).

No concerning multicollinearity was identified across the four logistic regression models (VIF range, 1.00–1.82; minimum tolerance, 0.55). The Hosmer–Lemeshow tests did not indicate statistically detectable lack of fit for Model 1 (chi-square = 10.62, df = 8, *p* = 0.224), Model 2 (chi-square = 13.18, df = 8, *p* = 0.106), Model 3 (chi-square = 9.21, df = 8, *p* = 0.325), or Model 4 (chi-square = 11.59, df = 8, *p* = 0.170). The corresponding AUCs were 0.717 (95% CI 0.668–0.768), 0.646 (95% CI 0.589–0.700), 0.797 (95% CI 0.746–0.845), and 0.758 (95% CI 0.652–0.847), respectively; Nagelkerke’s pseudo-R-squared values were 0.203, 0.086, 0.308, and 0.247. Overall, the diagnostics indicated low collinearity and no statistically detectable lack of fit, while discrimination ranged from modest to acceptable across the explanatory models.

### 3.5 Qualitative interview findings and quantitative–qualitative integration

Sixteen patients were interviewed: 12 had delayed standard care, 10 reported non-professional health information exposure, and 7 had received an amputation recommendation. Themes were integrated with the corresponding quantitative results in a joint display rather than reported as a separate parallel narrative (Table 1).

Across strands, the qualitative findings converged with and expanded the quantitative results. Painless or apparently small wounds lowered perceived urgency; non-professional information supplied plausible reasons to continue observation or self-treatment; and non-specialist encounters could postpone escalation even when patients had technically sought help. For amputation decisions, the interviews showed that the living-support score represented concrete anticipated consequences – dependence, caregiving burden, cost, and limited rehabilitation access – rather than a generalized fear of surgery. No major discordance between the quantitative and qualitative strands was identified, although qualitative accounts revealed mechanisms that the structured variables could not fully capture.

## 4 Discussion

### 4.1 Principal findings and significance

This study traced the pathway from first recognition of foot abnormalities to standard diabetic foot care and, for some patients, to amputation decision-making. Delayed standard care was common, but it was rarely a matter of complete inaction. In many cases, patients first used home observation, self-dressing changes, pharmacy medication, or repeated management in non-specialist facilities. Higher non-professional health information exposure scores and home-based or non-standard care were associated with delayed standard care, which was in turn associated with Wagner grade 3–5 disease at admission and amputation. Among patients who underwent amputation, delayed decision-making was also common, and greater concerns about post-amputation living support were independently associated with this delay. Taken together, these findings suggest that delayed treatment in diabetic foot ulcers reflects a combination of risk perception, information exposure, initial care behavior, family caregiving capacity, and expectations about life after amputation. By combining structured quantitative data with clinical outcomes and patient narratives, this study provides practical evidence for improving early risk recognition, standard care pathway education, correction of misleading health information, and amputation decision support.

### 4.2 Core mechanisms of delayed standard care: from symptom misjudgment to non-standard initial responses

Only a minority of patients directly received standard care after first noticing foot abnormalities, whereas a substantial proportion first received non-specialist medical care or used home-based or non-standard care. Thus, delayed standard care may be better understood as delayed escalation to appropriate care rather than a complete absence of care-seeking. Previous qualitative research has similarly described painless or apparently minor wounds, prior self-care experience, and initial consultation outside specialist services as reasons for postponing appropriate help-seeking (21). These parallels establish that symptom misjudgment is recurrent, while the integrated findings clarify how it can translate into delayed escalation. Early diabetic foot wounds may progress without pain, particularly in patients with peripheral neuropathy, so a small or painless lesion can reduce perceived susceptibility to deterioration and perceived disease severity. In Health Belief Model terms, familiar self-care or nearby non-specialist care may also appear to involve fewer perceived barriers than specialist assessment, whereas visible worsening, drainage, odor, or black discoloration may become a late cue to action (45). The quantitative and qualitative findings together support this sequence: patients acted, but their initial action was proportionate to a low perceived threat, and escalation often occurred only after the threat became unmistakable. Education should therefore do more than advise patients to seek care promptly; it should identify concrete warning signs, state that absence of pain is not evidence of mild disease, and specify where and how to obtain specialist diabetic foot care (1, 2, 15–21).

### 4.3 Non-professional health information exposure as a real-world factor in delayed standard care

Exposure to non-professional health information was frequent in this cohort, and higher exposure scores were independently associated with delayed standard care. Previous reviews have established that health misinformation on social media is common and may be anecdotal, fragmented, overly reassuring, or commercially driven (23–25, 32). Those studies identify the information problem but do not fully explain how exposure may be translated into delayed care in diabetic foot ulcers. From a health-literacy perspective, access to information is not equivalent to the ability to appraise source quality, reconcile competing claims, and apply information to a time-sensitive clinical problem (16, 17, 46). Wilson’s information-behavior model further suggests that information seeking under uncertainty is shaped by accessibility, social context, and perceived credibility, rather than professional expertise alone (47). In this setting, testimonials about topical remedies or limb salvage may confirm a preference to avoid hospitalization and make continued observation, self-treatment, or repeated non-specialist care appear reasonable. This provides a plausible explanation for the observed association without implying that information exposure necessarily caused delay; reverse or bidirectional relationships remain possible. Clinicians should therefore ask which sources influenced the patient’s decisions, and medical institutions should provide concise, credible, and actionable diabetic foot information through the channels that patients already use.

### 4.4 Delayed standard care, disease severity, and limb outcomes

The delayed standard care group had higher proportions of Wagner grade 3–5 disease, gangrene, and amputation, as well as longer hospital stays and higher hospitalization costs. Previous studies and reviews have also linked longer pre-hospital or referral pathways with more severe disease and poorer limb outcomes (10, 11, 42, 43). The present findings add a clinically plausible explanation for this relationship. During prolonged self-management or non-specialist care, ischemia may remain unrecognized, pressure may not be adequately offloaded, infection control and debridement may be incomplete, and necrotic tissue may progress. These missed or delayed components of standard care can permit wound enlargement, gangrene, deeper tissue involvement, and loss of limb-salvage options before multidisciplinary assessment (2, 4, 6–10). Infection did not differ significantly between groups. This may partly reflect its already high prevalence in the hospitalized cohort (68.5%), whereas Wagner grade and gangrene more directly capture accumulated depth and tissue destruction. The observational design cannot establish that delay caused amputation; severe disease may also influence care-seeking, and residual confounding remains. Nevertheless, the consistent direction of associations across the alternative time thresholds reduces concern that the findings depend entirely on the prespecified >7-day definition.

### 4.5 Delayed amputation decision-making: concerns about living support beyond fear of surgery

More than half of the patients with complete amputation decision-making data experienced delayed decision-making, and greater concerns about post-amputation living support remained associated with delay after adjustment. Previous qualitative and review evidence has described fear, loss of function, family burden, financial pressure, and uncertainty about rehabilitation as important elements of amputation decisions (26–31). The present mixed-methods findings extend that literature by showing both a measured association with delay and the practical reasoning underlying it. Interview participants were often less focused on the operation itself than on anticipated dependence, burdening family members, rehabilitation and prosthetic access, cost, and reduced social participation. In Health Belief Model terms, these anticipated consequences operate as perceived barriers that may outweigh the perceived benefit of timely surgery even when disease severity is understood (45). This explains why repeating medical risk information alone may not resolve hesitation. Communication should also make the post-discharge pathway concrete by addressing expected function, caregiver roles, rehabilitation timing, prosthetic options, insurance coverage, and the consequences of postponement; structured decision support and caregiver involvement may further reduce uncertainty (33, 34). This interpretation applies only to the conditional amputation subgroup and should not be generalized to all patients with diabetic foot ulcers.

### 4.6 Clinical implications and intervention recommendations

This study has several implications for diabetic foot management and public health education. First, an early warning-sign checklist should be provided to patients with diabetes and high-risk feet, emphasizing ulceration, black discoloration, purulent discharge, worsening redness and swelling, gangrene, poor healing after burns, and persistent wound enlargement as signs requiring urgent care (2, 5, 15–20). Second, outpatient and inpatient assessments should include questions about the pathway from first noticing foot abnormalities to seeking care, with particular attention to self-management, repeated non-specialist care, and prolonged observation. Third, education should address non-professional health information directly, helping patients distinguish credible medical advice from commercial, fragmented, or unverified treatment information (23–25, 32). Fourth, structured amputation decision support should be provided for patients of amputation or those for whom amputation has been explicitly recommended. Such support should cover the purpose of amputation, risks of delay, postoperative care, rehabilitation resources, prosthetic support, and family care arrangements (26, 27, 29, 33, 34). Fifth, family members or primary caregivers should be included in diabetic foot treatment and amputation decision-making discussions so that patients can understand medical risks while also receiving realistic expectations about support after treatment.

### 4.7 Limitations

This study has limitations. First, it was conducted at one center in one country. Differences in referral systems, digital information environments, family caregiving, rehabilitation access, prosthetic services, and insurance may limit international generalizability. Second, symptom onset, early response pathways, and exposure to non-professional health information partly relied on recall. Patient reports, family information, and medical records were used to cross-check key events, but recall bias could not be eliminated. Third, interviewer-administered questions about non-standard care and treatment decisions may have been affected by social desirability bias. Fourth, although EFA provided preliminary support for a one-factor structure for each researcher-designed item set, both analyses were conducted in the same samples used for the main association analyses, and the living-support item set was evaluated in only 102 participants. Independent confirmatory factor analysis and assessment of measurement invariance are therefore required before these item sets can be regarded as fully validated scales. Fifth, complete-case analysis may have introduced selection bias, and missingness was not assumed to be completely at random. Sixth, the observational design cannot establish causality; reciprocal relationships and residual confounding remain possible. Seventh, because all patients with a documented amputation recommendation ultimately underwent the procedure, the subgroup findings characterize delayed decision-making before eventual acceptance and cannot be generalized to patients who persistently decline recommended amputation or leave care without undergoing the procedure. Finally, although the qualitative interviews reached thematic saturation, the sample was small and was intended to explain the quantitative findings rather than estimate population frequencies.

## 5 Conclusion

This study showed that delayed entry into standard care was common among patients with diabetic foot ulcers and was associated with non-standard initial response pathways, greater exposure to non-professional health information, more advanced disease severity, and amputation. Although all patients with a documented amputation recommendation ultimately underwent the procedure, delayed acceptance was frequent among those with complete decision-making data and was associated with greater concerns about post-amputation living support. These findings support interventions that combine early warning-sign recognition, clear guidance on standard care pathways, critical appraisal of non-professional health information, and structured amputation decision support addressing rehabilitation, caregiving, financial, and post-discharge needs. Multicenter studies and further validation of the researcher-designed indices are needed before broader generalization.

## Data Availability

The raw data supporting the conclusions of this article will be made available by the authors, without undue reservation.
