# Supplementary material for: Delayed standard care in diabetic foot ulcers: the roles of initial response pathways and non-professional health information
Source: Front Public Health. 2026 Sep 25;14:1905489. doi: 10.3389/fpubh.2026.1905489 (PMC13650000; doi:10.3389/fpubh.2026.1905489)
Supplement: Supplementary file 1 [file Table_1.docx]

**Supplementary Table 1. Sensitivity analyses using alternative thresholds for delayed standard care**

*Alternative definitions: delayed standard care defined as ≥24 h and severe delayed standard care defined as >30 days*

| **Model** | **Outcome / exposure definition** | **Key factor** | **OR** | **95% CI** | **P value** |
| --- | --- | --- | --- | --- | --- |
| Model 1a | Delayed standard care defined as ≥24 h | Non-specialist medical care vs direct standard care | 1.82 | 0.96–3.45 | 0.07 |
|  |  | Home-based or non-standard care vs direct standard care | 3.76 | 1.98–7.14 | <0.001 |
|  |  | Non-professional health information exposure score, per 1-point increase | 1.42 | 1.16–1.74 | 0.001 |
| Model 1b | Severe delayed standard care defined as >30 days | Non-specialist medical care vs direct standard care | 2.58 | 1.24–5.36 | 0.01 |
|  |  | Home-based or non-standard care vs direct standard care | 6.12 | 2.89–12.96 | <0.001 |
|  |  | Non-professional health information exposure score, per 1-point increase | 1.92 | 1.52–2.42 | <0.001 |
| Model 2a | Wagner grade 3–5 | Delayed standard care defined as ≥24 h | 1.86 | 1.19–2.91 | 0.006 |
| Model 2b | Wagner grade 3–5 | Severe delayed standard care defined as >30 days | 2.74 | 1.58–4.75 | <0.001 |
| Model 3a | Amputation | Delayed standard care defined as ≥24 h | 1.62 | 0.98–2.68 | 0.06 |
| Model 3b | Amputation | Severe delayed standard care defined as >30 days | 2.31 | 1.34–3.98 | 0.002 |

OR, odds ratio; CI, confidence interval.

Model 1a and Model 1b assessed factors associated with delayed standard care using alternative thresholds. Models 2a/2b and 3a/3b assessed associations between alternative delay definitions and Wagner grade 3–5 disease and amputation, respectively.

Models were adjusted for the same covariates as the corresponding primary multivariable logistic regression models.
